# Species diversity and community structure of crustacean zooplankton in the highland small waterbodies in Northwest Yunnan, China

**DOI:** 10.7717/peerj.12103

**Published:** 2021-09-02

**Authors:** Xing Chen, Qinghua Cai, Lu Tan, Shuoran Liu, Wen Xiao, Lin Ye

**Affiliations:** 1 State Key Laboratory of Freshwater Ecology and Biotechnology, Institute of Hydrobiology, Chinese Academy of Sciences, Wuhan, Hubei, China; 2 University of Chinese Academy of Sciences, Beijing, China; 3 Institute of Eastern-Himalaya Biodiversity Research, Dali University, Dali, Yunnan, China

**Keywords:** Northwest Yunnan, Small waterbodies, Biodiversity, Crustacean zooplankton

## Abstract

Small waterbodies are a unique aquatic ecosystem with an increasing recognition for their important role in maintaining regional biodiversity and delivering ecosystem services. However, small waterbodies in Northwest Yunnan, one of the most concerned global biodiversity hot-spots, remain largely unknown. Here, we investigated the community structure of crustacean zooplankton and their relationships with limnological, morphometric and spatial variables in the highland small waterbodies in Northwest Yunnan in both the dry (October 2015) and rainy (June 2016) seasons. A total of 38 species of crustacean zooplankton were identified in our study, which is significantly higher than many other reported waterbodies in the Yunnan–Guizhou plateau as well as in the Yangtze River basin. This suggests that the highland small waterbodies are critical in maintaining regional zooplankton diversity in Northwest Yunnan. Meanwhile, we found limnological variables could explain most variation of crustacean zooplankton community, comparing to the morphometric and spatial variables in both the rainy and dry seasons. Our study revealed the diversity and community structure of crustacean zooplankton in the highland small waterbodies in Northwest Yunnan and highlighted the importance of small waterbodies in maintaining regional biodiversity.

## Introduction

Small waterbodies are critical for regional biodiversity and are increasingly recognized for their essential role in maintaining biodiversity and providing ecosystem services (Williams et al., 2004; Biggs, von Fumetti & Kelly-Quinn, 2017; Kuczyńska-Kippen, 2020). Small waterbodies with low density or without fish and abundant submerged vegetation support high biodiversity of aquatic organisms and contributed a large proportion of rare or endemic species to local freshwater habitats (Williams et al., 2004; Oertli et al., 2005; Scheffer et al., 2006). Also, small waterbodies have important ecological functions (Céréghino et al., 2014; Biggs, von Fumetti & Kelly-Quinn, 2017). Small waterbodies can significantly reduce nutrient concentrations and protect downstream waters (Cheng & Basu, 2017). On the other hand, small waterbodies are vulnerable to environmental changes because of their small size (Biggs, von Fumetti & Kelly-Quinn, 2017).

Crustacean zooplankton is an important group in freshwater ecosystems because they occupy central positions in aquatic food webs, transferring energy to higher trophic levels (Sommer et al., 1986; Fussmann, 1996). In addition, crustacean zooplankton is sensitive to climate and environmental change (Keller & Conlon, 1994; Shurin et al., 2010; Jones & Gilbert, 2016). For quite a long time, the research on crustacean zooplankton in freshwater ecosystems has been mainly focused on lakes (Barbiero et al., 2019) and reservoirs (Liu et al., 2020). Yet, the ecology of crustacean zooplankton in highland small waterbodies remains seldom addressed.

Northwest Yunnan, located in Southwest China, has been designated as a global biodiversity “hot-spot” by World Wildlife Fund (WWF) and International Union for Conservation of Nature (IUCN) because of its rich biodiversity, unique and diverse highland landscape (Mackinnon et al., 1996; Xu & Wilkes, 2004; Trizzino et al., 2014). This region is in the upper stream of the Yangtze (Jinsha) River, the Mekong (Lancang) River, the Salween (Nujiang) River, and the Irrawaddy (Dulongjiang) River, attracting extensive attention of local and international communities (Xu & Wilkes, 2004; Ao et al., 2021). Currently, most ecology and biodiversity related studies in this region focus on the terrestrial vegetation and endangered wild animals (Xu & Wilkes, 2004; Li et al., 2014), yet still few studies addressed the aquatic ecosystems, especially for small waterbodies ecosystems.

In this study, we focus on the community structure and species diversity of crustacean zooplankton in highland small waterbodies in Northwest Yunnan, China. Besides the limnological variables (*e.g.*, water temperature, nutrients), previous studies have reported that morphometric variables (*e.g.*, surface area, depth) and spatial variables (*e.g.*, distance) also have critical effects on zooplankton diversity and community composition (Dodson, 1992; Beisner et al., 2006; MacLeod, Keller & Paterson, 2018). Here, we hypothesized that crustacean zooplankton in the small waterbodies are co-determined by limnological, morphometric, and spatial variables. Specifically, the main aims of our study are to understand: (i) the diversity and community structure of crustacean zooplankton in highland small waterbodies in Northwest Yunnan, (ii) the difference of community structure in rainy and dry seasons, (iii) how the limnological, morphometric and spatial variables determine the spatiotemporal variations of diversity and community structure.

## Materials & methods

### Study sites and field sampling

**Figure 1:**
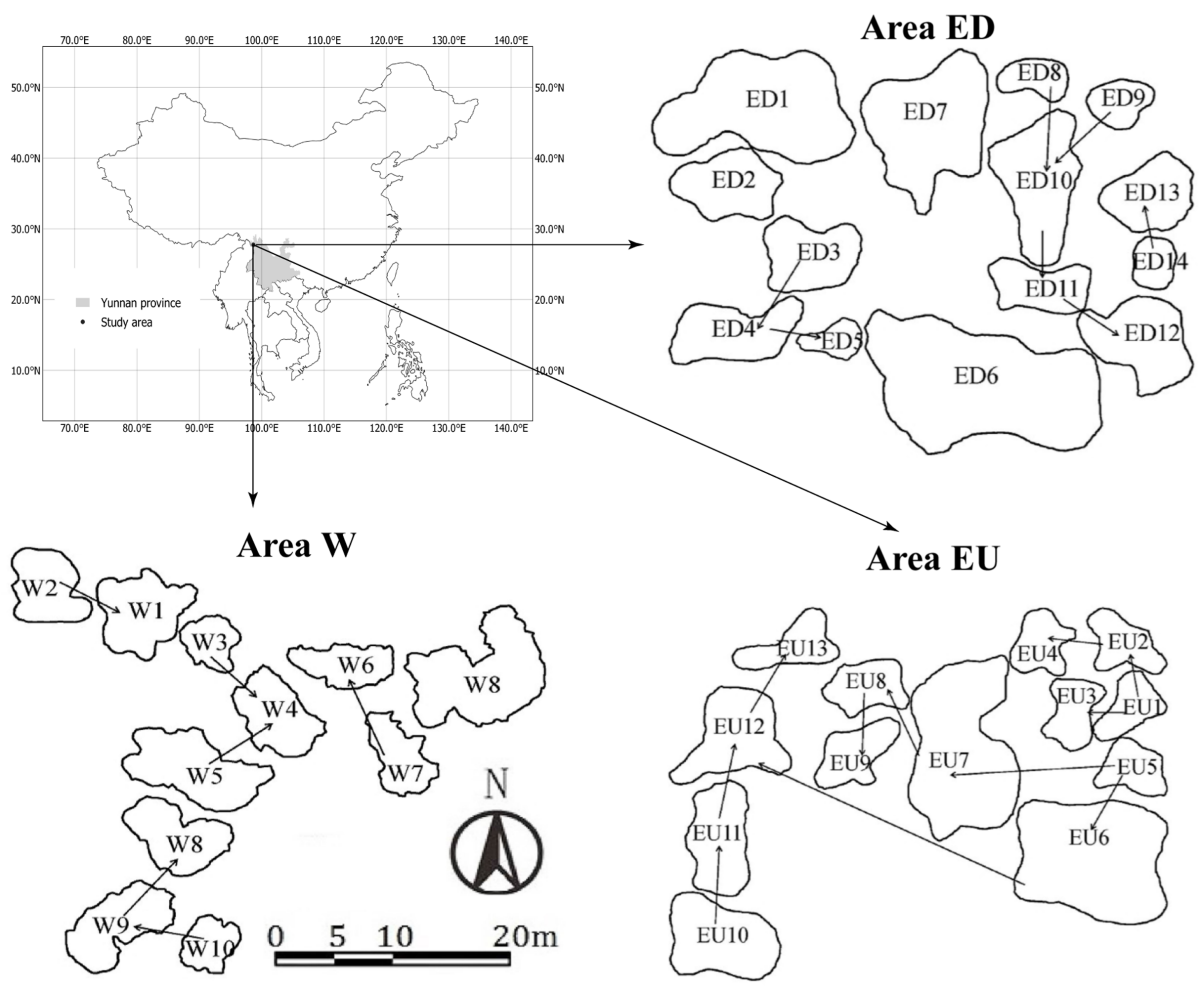
Sampling areas and spatial distribution of small waterbodies in Gongshan County, Yunnan province, China. The arrows represent the connectivity and water flow direction.

The study sites were distributed on the east (Area E) and west (Area W) sides of a high mountain ridge 3,700 m in Gong-shan Country, Yunnan province, China (Fig. 1). The average annual temperature and precipitation (from August 1, 2014 to July 31 2015) was 7.7 °C and 2,515 mm respectively (Liu et al., 2018). There was a disused road lying across the “Area E”, which separated this area into Upstream (EU) and Downstream (ED) subgroups. The average elevation and area of small waterbodies are 3,131 m and 9.9 m^2^ for the area W, 3,328 m and 13 m^2^ for the area EU, and 3,274 m and 41 m^2^ for the area ED, respectively. In addition, these small waterbodies have no fish, but have abundant macrophytes.

A total of two samplings were carried out in the dry (October 2015) and rainy (June 2016) seasons reflecting different hydrological regimes. A total of 30 and 32 small waterbodies were sampled in the dry and rainy seasons, respectively. For each small waterbody, the morphometric and spatial variables, including the water depth, water surface area, coordinates and altitude (using Garmin eTerx20, China) were measured. The physical parameters, including conductivity (Cond), dissolved oxygen (DO), pH, and water temperature (WT), were measured by a portable multi-parameter device (YSI Professional plus, Yellow Springs, OH, USA). Water samples for chemical analysis were collected from the center of each small waterbodies using a 350 ml plastic bottle. Ammonia nitrogen (NH_3_N), nitrate nitrogen (NO_3_N), total nitrogen (TN), phosphate (PO_4_P), total phosphorus (TP), dissolved silicate (DSi), and dissolved organic carbon (DOC) were analyzed by segmented flow analyzer (Skalar SAN++; Breda, Netherlands), according to the user manual. Also, another 350 ml water sample was filtered through a micro-filter (~1.2 µm, GF/C Whatman, Maidstone, UK) for the measurement of chlorophyll a (Chl-a). The concentration of Chl-a was measured with a spectrophotometer (Shimadzu UV-1800, Kyoto, Japan) with the standard method of APHA (1999).

Crustacean zooplankton samples were collected with a plankton net (64 µm in mesh size) by filtering 20 L water sampled from the open water region in each small waterbody. All crustacean zooplankton samples were preserved with 5% formalin immediately.

### Zooplankton counting and identification

Crustacean zooplankton was counted and identified under the stereoscope (Zeiss Stereo Discovery V20, Oberkochen, German). All crustacean zooplankton samples were identified to the species level as far as possible. Specifically, all samples were screened under the stereoscope because of the low density of the crustacean zooplankters. The major reference books for identification were Chiang & Du (1979), Shen (1979) and Błędzki and Rybak (2016).

### Statistical analysis

A rarefaction was used to compare species richness and Shannon diversity between the rainy season and dry season because biodiversity was affected by sampling efforts, such as the number of sites and individual numbers (Chao et al., 2014). Specifically, we calculated species richness and Shannon diversity index for the whole waterbodies (Chao et al., 2014). Then, we plotted individual-based rarefaction curves for each season to compare the differences of species richness and Shannon diversity index.

A nonmetric multidimensional scaling (NMDS) was carried out to illustrate taxonomic and abundance similarity between the rainy and dry seasons. Further, the similarity percentage analysis (SIMPER) was conducted to investigate differences in community composition between the rainy and dry seasons and to determine the contribution of each species to the Bray–Curtis dissimilarities (Clarke, 1993).

In order to test our hypothesis, we conducted the variation partitioning with redundancy analysis (RDA) to compare species composition variation with the limnological, morphometric, and spatial variables. To avoid collinearity, only limnological variables with the correlation coefficient below 0.7 were selected as predictor variables (Dormann et al., 2013). As a result, the limnological variables, including TN, NO_3_N, NH_3_N, PO_4_P, DSi, DOC, Cond, WT, Chl-a, were kept for further RDA. Water depth and surface area were selected as morphometric variables. Spatial variables can reflect the community dispersal limitation according to the metacommunity theory (Heino et al., 2017). The candidate spatial variables for the RDA were determined by Moran Eigenvector Maps (MEMs) (Borcard & Legendre, 2002). First, longitude and latitude were converted into Cartesian coordinates (the unit is kilometer). Second, the Euclidian distance matrix among the small waterbodies was calculated. Then, five eigenvectors with positive eigenvalues in MEMs were determined as the spatial predictors for RDA.

In the RDA, forward selection method was used to select the key variables explaning the variation of the crustacean zooplankton community (Blanchet, Legendre & Borcard, 2008). To reduce the weight of species abundance, abundance data were Hellinger transformed before variation partitioning (Legendre & Gallagher, 2001). Finally, five limnological variables (NO_3_N, DSi, Cond, WT and DO), two morphometric variables (water depth and surface area) and four spatial variables (MEM1, MEM2, MEM3 and MEM5) were selected in variation partitioning (Table S2). All analyses were implemented with R statistical software (R Development Core Team, 2020). Rarefaction was carried out with “*iNEXT*” package (Hsieh, Ma & Chao, 2016). MEMs and RDA variation partitioning were performed using “*vegan*” package (Oksanen et al., 2019).

## Results

### Community composition

A total of 38 crustacean zooplankton taxa, including 20 Cladocera and 18 Copepoda species, were identified (Table 1). In the rainy season, the most common species were *Cyclops vicinus*, *Mesocyclops leuckarti*, *Alona affinis*, *Microclops varicaricans*, *Moina irrasa, Cyclops strenuuss*, *Ectocyclops phaleratus*, which occurred in more than 50% of the surveyed small waterbodies. In the dry season, *Chydorus ovalis*, *M*. *varicaricans*, *Tropocyclops prasinus*, *Ceriodaphnia laticaudata*, *Alonella exigua*, had a relative occurrence above 50% (Table 1).

**Table 1:** Relative occurrences of crustacean zooplankton species in all samples, samples in area E, and samples in area W in the rainy (32 samples) and dry (30 samples) seasons.

Species	Rainy season			Dry season		
	% of all samples	% of E samples	% of W samples	% of all samples	% of E samples	% of W samples
*Alona affinis*	65.6	72.7	50.0	0.0	0.0	0.0
*Moina irrasa*	56.3	54.5	60.0	0.0	0.0	0.0
*Chydorus ovalis*	40.6	31.8	60.0	93.3	90.1	100.0
*Diaphanosoma sp*.	31.3	31.8	30.0	12.5	9.9	0.0
*Bosmina coregoni*	21.9	27.3	10.0	3.3	4.5	0.0
*Alona guttata*	12.5	18.2	0.0	3.3	4.5	0.0
*Ceriodaphnia laticaudata*	0.0	0.0	0.0	53.3	45.5	75.0
*Alonella exigua*	0.0	0.0	0.0	53.3	59.1	37.5
*Alona karua*	0.0	0.0	0.0	30.0	31.8	25.0
*Graptoleberis testudinaria*	0.0	0.0	0.0	20.0	18.2	25.0
*Alona rectangula*	0.0	0.0	0.0	23.3	22.7	25.0
*Moina rectirostris*	0.0	0.0	0.0	16.7	13.6	25.0
*Ceriodaphnia quadrangula*	0.0	0.0	0.0	20.0	9.1	50.0
*Alonella globulosa*	0.0	0.0	0.0	16.7	13.6	25.0
*Ceriodaphnia reticulata*	0.0	0.0	0.0	13.3	18.2	0.0
*Alona quadrangularis*	0.0	0.0	0.0	10.0	13.6	0.0
*Chydorus barroisi*	0.0	0.0	0.0	6.7	9.1	0.0
*Alonella sp*.	0.0	0.0	0.0	6.7	9.1	0.0
*Alona sp*.	0.0	0.0	0.0	3.3	0.0	12.5
*Alonella nana*	0.0	0.0	0.0	3.3	0.0	12.5
*Cyclops vicinus*	71.2	63.6	90.0	0.0	0.0	0.0
*Mesocyclops leuckarti*	71.2	63.6	90.0	0.0	0.0	0.0
*Microclops varicaricans*	62.5	50.0	90.0	90.0	86.4	100.0
*Ectocyclops phaleratus*	59.4	38.5	90.0	6.7	9.1	0.0
*Cyclops strenuuss*	56.3	45.5	80.0	3.3	0.0	12.5
*Limnoithona sinensis*	46.7	36.4	80.0	26.7	4.5	87.5
*Nitocra lacustri*	43.8	54.5	20.0	0	0	0
*Sinodiaptomus sarsi*	43.8	31.8	70.0	0	0	0
*Eucyclops serrulatus*	37.5	40.9	30.0	16.7	22.7	0.0
*Sinocalanus dorrii*	21.9	13.6	40.0	10.0	4.5	25.0
*Onychocamptus mohammed*	21.9	27.3	10.0	46.7	27.3	100.0
*Neutrodiaptomus mariadvigae*	15.6	13.6	20.0	10.0	0.0	37.5
*Bryocamptus sp*.	9.4	13.6	0.0	3.3	4.5	0.0
*Tropodiaptomus hebereri*	6.3	9.1	0.0	13.3	0.0	50.0
*Tropocyclops prasinus*	0.0	0.0	0.0	66.7	63.6	75.0
*Paracyclops fimbriatus*	0.0	0.0	0.0	16.7	18.2	12.5
*Paracyclops affinis*	0.0	0.0	0.0	10.0	9.1	12.5
*Schmackeria inopinus*	0.0	0.0	0.0	10.0	4.5	25.0

**Figure 2:**
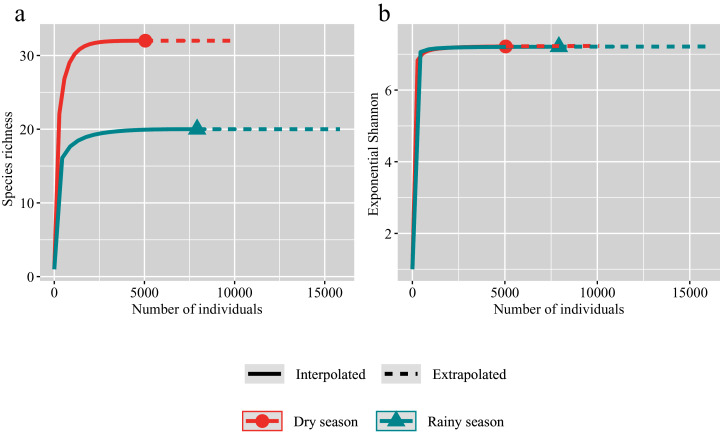
Individual-based rarefaction for the dry (red) and rainy (green) seasons. Symbols represent species richness (A) and Exponential Shannon (B). Continuous lines refer to interpolation, dotted lines refer to extrapolation.

The species accumulation curves showed that we have sampled considerable individuals in both the rainy and dry seasons (Fig. 2). The observed species richness is almost same as the estimated values of species richness in both the rainy and dry seasons. And the species richness in the dry season is significantly higher than that in the rainy season (Fig. 2A). However, Shannon diversity index showed that an explicit overlapping of observed and estimated species richness for the rainy and dry seasons (Fig. 2B).

**Figure 3:**
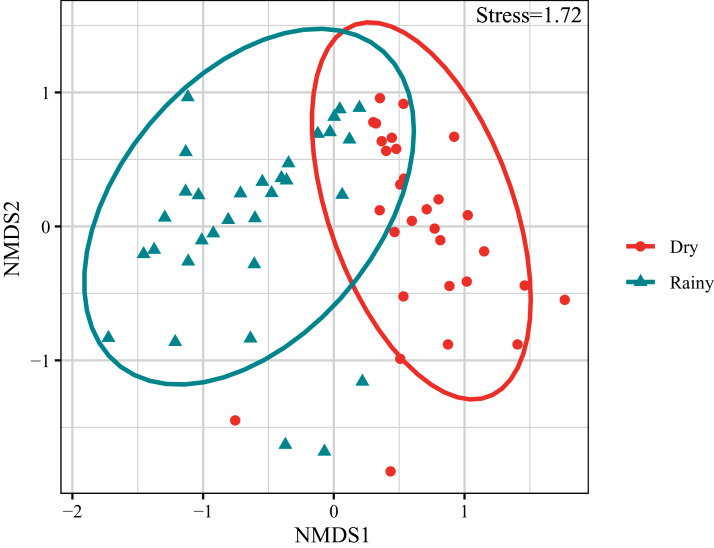
Non-metric multidimensional scaling ordination (NMDS) of crustacean zooplankton communities.

The composition and abundance of crustacean zooplankton changed significantly between the rainy and dry seasons., *M. varicaricans*, *C. ovalis*, *C. vicinus*, *A. exigua* and *S. sarsi* are most influential species based on cumulative contribution (Table 2). Further, species compositions differed significantly between the rainy and dry seasons (Fig. 3).

**Table 2:** Results of SIMPER analysis for species that accounted for the 90% of cumulative contribution.

Species	Average dissimilarity	Standard deviation	Ratio	Cumulative contribution	*P*
*M. leuckarti*	0.20879	0.20616	1.0127	0.2313	0.675
*M. varicaricans*	0.15750	0.16750	0.9403	0.4058	**0.001**
*C. ovalis*	0.10450	0.14320	0.7297	0.5216	**0.001**
*C. vicinus*	0.07331	0.09875	0.7424	0.6028	**0.001**
*A. exigua*	0.06395	0.14393	0.4443	0.6737	**0.019**
*S. sarsi*	0.04930	0.12002	0.4108	0.7283	0.091
*E. phaleratus*	0.02948	0.04346	0.6783	0.7610	**0.006**
*C. laticaudata*	0.02447	0.05694	0.4299	0.7881	**0.016**
*L. sinensis*	0.02324	0.04526	0.5136	0.8139	0.600
*C. strenuuss*	0.02290	0.04765	0.4806	0.8392	**0.034**
*M. irrasa*	0.01874	0.03545	0.5286	0.8600	**0.008**
*E. serrulatus*	0.01756	0.04665	0.3765	0.8794	0.464
*A. affinis*	0.01276	0.01787	0.7145	0.8936	**0.001**

**Note:**

Bold values indicate statistical significance at the *p* < 0.05 level.

### Crustacean zooplankton community variation partitioning

**Figure 4:**
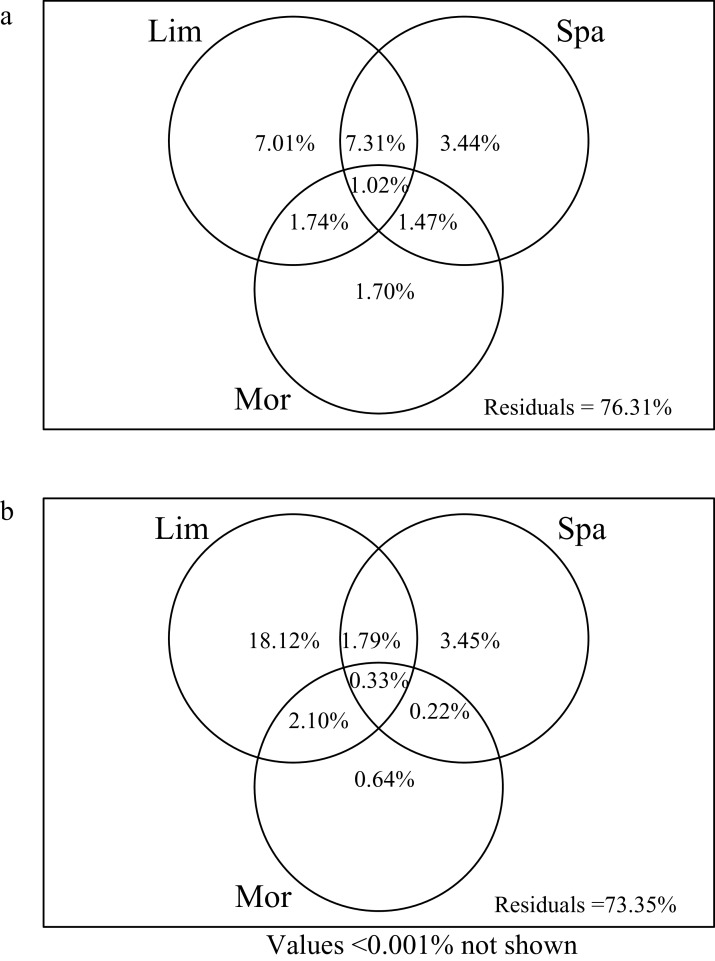
Venn diagram representing the variation partitioning of crustacean zooplankton community composition explained by explanatory variable. (Lim) Limnological variables. (Spa) spatial variables represented by principal coordinates of neighbour matrices. (Mor) morphometric variables. (A) Dry season. (B) Rainy season.

Limnological variables explained the most variation of crustacean zooplankton community in both the rainy (NO_3_N, DSi, Cond and DO) and dry (NO_3_N and WT) seasons, compared to the morphometric and spatial variables (Fig. 4). In the dry season, the limnological, morphometric, and spatial variables explained 23.69% of the crustacean zooplankton community structure (Fig. 4A). The limnological variables explained the most variation of zooplankton community structure (7.01%), which is significantly higher than spatial variables (3.44%) and morphometric variables (1.70%). Variation partitioning revealed 7.31% of the shared variation between limnological variables and spatial variables. However, only 1.48% of the variation was shared between the morphometric and spatial variables.

In the rainy season, all predictors explained 26.65% of the crustacean zooplankton community structure (Fig. 4B), which was slightly higher than the dry season. The limnological variables alone explained 18.12% of the variation. And the spatial variables had a lower contribution (3.45%) and followed by morphometric variables (0.64%).

## Discussion

One interesting finding of our study is that the species richness in our study area is significantly higher than many other reported waterbodies in the Yunnan–Guizhou plateau as well as in the Yangtze River basin (Table 3). For example, Guo et al. (2009) identified 36 crustacean zooplankton species in 13 different lakes in the Yunnan–Guizhou plateau with areas ranged from 10.7 to 297.9 km^2^. Another similar research carried out in the plateau lake (Erhai Lake) in Yunnan province only recorded 11 crustacean zooplankton species for 12 field stations with 1-year continuous monthly monitoring (Yang et al., 2014). Comparing to the lakes and other waterbodies, small waterbodies usually have a high habitat heterogeneity which can support more diverse species and maintain a high diversity community (Williams et al., 2004).

**Table 3:** A comparison of the species richness in the small waterbodies in the highland small waterbodies in Northwest Yunnan with other waterbodies in Yunnan-Guizhou plateau and Yangtze River basin.

Study area	Province	Area (km^2^)	Species richness	Reference
Thirteen lakes in Yunnan and Guizhou (*n* = 112)	Yunnan and Guizhou	10.7~297.9	36	Guo et al., 2009
Gaoyou Lake (*n* = 26)	Jiangsu	674	26	Wei et al., 2017
Chaohu Lake (*n* = 228)	Anhui	780	23	Deng et al., 2008
Lugu Lake (*n* = 36)	Yunnan	57.7	23	Dong & Wang, 2014
Fuxian Lake (*n* = 220)	Yunnan	211	8	Pan et al., 2009
Erhai (*n* = 144)	Yunnan	249	11	Yang et al., 2014
Our study (*n* = 62)	Yunnan	<0.001	38	Our study

**Note:**

*n* indicating the number of total samples in the reported case.

The absence of predatory fish and complex habitat with abundant macrophytes might explain high crustacean zooplankton diversity in the highland small waterbodies in our study. Fish is more likely to be absent in small and isolated waterbodies because of high risks of extinction and low chances of colonization (Scheffer et al., 2006). In our field survey, we did not observe fish in any waterbodies. Presence of fish could profoundly impact crustacean zooplankton community structure by reducing species richness and simplifying community composition, especially in small waterbodies (Scheffer et al., 2006). The predation from fish is an important factor affecting crustacean zooplankton in small lakes (Pinel-Alloul & Mimouni, 2013). Meanwhile, some studies also suggested that macrophyte cover is important to maintain zooplankton diversity because of macrophyte provide good habitats for zooplankton (Celewicz-Goldyn & Kuczynska-Kippen, 2017). These natural, temporal, and mountain small waterbodies have good water quality and high coverage of macrophytes (Kuczyńska-Kippen, 2020), providing ecological niches for rare (such as *Graptoleberis testudinaria* and *A. karua*) or endemic species (such as *T. hebereri* and *N. mariadvigae*).

The species compositions in the rainy and dry seasons are quite different in the highland small waterbodies in Northwest Yunnan, China. We found crustacean zooplankton richness was higher in the dry season compared to the rainy season. Higher richness in the dry season has also been reported in other studies and was associated with higher environmental heterogeneity and longer water residence time (Pourriot, Rougier & Miquelis, 1997*; Melo & Medeiros, 2013*), allowing more species to colonize in the small waterbodies. In terms of the species composition, *C*. *ovalis*, *M*. *varicaricans*, *T*. *prasinus*, *C*. *laticaudata*, *A*. *exigua* were the most common species in the dry season. However, in the rainy season, the common species shifted to *C*. *vicinus*, *M*. *leuckarti*, *A*. *affinis*, *M*. *varicaricans*, *M*. *irrasa, C*. *strenuuss*, *E*. *phaleratus*. Among these species, we found two endemic species (*Tropodiaptomus hebereri* and *Neutrodiaptomus mariadvigae*) in the Yunnan–Guizhou plateau (Shen, 1979). Also, we found nine common species (*e.g., C*. *vicinus*, *M. leuckarti*, *A*. *affinis*, *M*. *irrasa*) in the Yangtze River basin (Chiang & Du, 1979).

Our study also showed limnological variables explained most variation of crustacean zooplankton community in both the rainy and dry seasons, compared to the morphometric and spatial variables. This result is coherent with many other studies which also showed limnological variables as the most important factors in explaining variations of crustacean zooplankton compared to spatial variables. (Heino et al., 2017; Lévesque et al., 2017; Brasil et al., 2020). Our finding suggests that environmental filter played a key role in community structure in the highland small waterbodies in Northwest Yunnan, possibly related to their environmental heterogeneity. Previous experience showed that the environmental heterogeneity of small waterbodies in the Northwest of Yunnan depended on the watershed and precipitation (Liu et al., 2018).

We should add a caveat that not all potential limnological variables affecting the crustacean zooplankton communities were examined in our study due to limited data. Some researches suggested that macrophytes cover is important to maintain zooplankton diversity because macrophyte provide shelter from predators (Cazzanelli, Warming & Christoffersen, 2008; Sagrario et al., 2009). In our study, we did not address the effects of macrophytes. However, the zooplankton samples were collected in the open water area with no macrophytes, suggesting the direct effect of macrophytes on zooplankton samples was weak. Future works on factor shaping zooplankton community in small waterbodies could focus on the effect of macrophyte, which are probably important to affect zooplankton species assemblages (Celewicz-Goldyn & Kuczynska-Kippen, 2017).

## Conclusions

In this study, we reported the crustacean zooplankton community and their relationships with the limnological, morphometric and spatial variables in the highland small waterbodies in Northwest Yunnan for both the rainy and dry seasons. We identified 38 species of crustacean zooplankton, which is significantly higher than many other waterbodies in the Yunnan–Guizhou plateau as well as in the Yangtze River basin. This suggests that small waterbodies are biodiversity hotspot and are important in maintaining regional zooplankton diversity in Northwest Yunnan. Limnological variables could explain the most variation of crustacean zooplankton community, comparing to morphometric and spatial variables in both the rainy and dry seasons. This study improved our understanding of the diversity and community structure of crustacean zooplankton in the highland small waterbodies in Northwest Yunnan and highlighted the importance of small waterbodies for biodiversity conservation and research.

## Supplemental Information

10.7717/peerj.12103/supp-1 Supplemental Information 1 Environmental factors in rainy and dry season.

10.7717/peerj.12103/supp-2 Supplemental Information 2 Abundance of crustacean zooplankton in rainy and dry season.

10.7717/peerj.12103/supp-3 Supplemental Information 3 Mean values, maximum and minimum for limnological and morphometric variables in different season.

10.7717/peerj.12103/supp-4 Supplemental Information 4 Variables selected by forwarding selection in explaining variations of crustacean zooplankton communities.

## Additional Information and Declarations

### Data Availability

The following information was supplied regarding data availability: The raw data are available in the Supplemental Files.

## References

[ref-1] Ao SC, Chiu MC, Li XF, Tan L, Cai QH, Ye L (2021). Watershed farmland area and instream water quality co-determine the stream primary producer in the central Hengduan Mountains, Southwestern China. Science of the Total Environment.

[ref-2] APHA (1999). Standard methods for the examination of waste water.

[ref-53] Barbiero RP, Rudstam LG, Watkins JM, Lesht BM (2019). A cross-lake comparison of crustacean zooplankton communities in the Laurentian Great Lakes, 1997–2016. Journal of Great Lakes Research.

[ref-3] Beisner BE, Peres-Neto PR, Lindström ES, Barnett A, Longhi ML (2006). The role of environmental and spatial processes in structuring lake communities from bacteria to fish. Ecology.

[ref-4] Biggs J, von Fumetti S, Kelly-Quinn M (2017). The importance of small waterbodies for biodiversity and ecosystem services: implications for policy makers. Hydrobiologia.

[ref-6] Blanchet FG, Legendre P, Borcard D (2008). Forward selection of explanatory variables. Ecology.

[ref-7] Błędzki LA, Rybak JI (2016). Freshwater crustacean zooplankton of Europe.

[ref-8] Borcard D, Legendre P (2002). All-scale spatial analysis of ecological data by means of principal coordinates of neighbour matrices. Ecological Modelling.

[ref-9] Brasil J, Santos JBO, Sousa W, Menezes RF, Huszar VLM, Attayde JL (2020). Rainfall leads to habitat homogenization and facilitates plankton dispersal in tropical semiarid lakes. Aquatic Ecology.

[ref-10] Cazzanelli M, Warming TP, Christoffersen KS (2008). Emergent and floating-leaved macrophytes as refuge for zooplankton in a eutrophic temperate lake without submerged vegetation. Hydrobiologia.

[ref-11] Celewicz-Goldyn S, Kuczynska-Kippen N (2017). Ecological value of macrophyte cover in creating habitat for microalgae (diatoms) and zooplankton (rotifers and crustaceans) in small field and forest water bodies. PLOS ONE.

[ref-12] Céréghino R, Boix D, Cauchie HM, Martens K, Oertli B (2014). The ecological role of ponds in a changing world. Hydrobiologia.

[ref-13] Chao A, Gotelli NJ, Hsieh TC, Sander EL, Ma KH, Colwell RK, Ellison AM (2014). Rarefaction and extrapolation with Hill numbers: a framework for sampling and estimation in species diversity studies. Ecological Monographs.

[ref-14] Cheng FY, Basu NB (2017). Biogeochemical hotspots: role of small water bodies in landscape nutrient processing. Water Resource Research.

[ref-15] Chiang SC, Du NS (1979). Fauna sinica, crustacean: freshwater cladocera.

[ref-16] Clarke KR (1993). Non-parametric multivariate analyses of changes in community structure. Australian Journal of Ecology.

[ref-17] Deng D, Xie P, Zhou Q, Yang H, Guo L, Geng H (2008). Field and experimental studies on the combined impacts of cyanobacterial blooms and small algae on crustacean zooplankton in a large, eutrophic, subtropical, Chinese lake. Limnology.

[ref-18] Dodson S (1992). Predicting crustacean zooplankton species richness. Limnology and Oceanography.

[ref-19] Dong Y, Wang Z (2014). Zooplankton community structure and its seasonal variation in the surface water of Lugu Lake. Journal of Hydreoecology.

[ref-20] Dormann CF, Elith J, Bacher S, Buchmann C, Carl G, Carré G, Marquéz JRG, Gruber B, Lafourcade B, Leitão PJ, Münkemüller T, McClean C, Osborne PE, Reineking B, Schröder B, Skidmore AK, Zurell D, Lautenbach S (2013). Collinearity: a review of methods to deal with it and a simulation study evaluating their performance. Ecography.

[ref-21] Fussmann G (1996). The importance of crustacean zooplankton in structuring rotifer and phytoplankton communities: an enclosure study. Journal of Plankton Research.

[ref-23] Guo N, Zhang M, Yu Y, Qian S, Li D, Kong F (2009). Crustacean zooplankton communities in 13 lakes of Yunnan–Guizhou plateau: Relationship between crustacean zooplankton biomass or size structure and trophic indicators after invasion by exotic fish. Annales de Limnologie.

[ref-24] Heino J, Soininen J, Alahuhta J, Lappalainen J, Virtanen R (2017). Metacommunity ecology meets biogeography: effects of geographical region, spatial dynamics and environmental filtering on community structure in aquatic organisms. Oecologia.

[ref-25] Hsieh TC, Ma KH, Chao A (2016). iNEXT: an R package for rarefaction and extrapolation of species diversity (Hill numbers). Methods in Ecology and Evolution.

[ref-26] Jones NT, Gilbert B (2016). Changing climate cues differentially alter zooplankton dormancy dynamics across latitudes. Journal of Animal Ecology.

[ref-27] Keller W, Conlon M (1994). Crustacean zooplankton communities and Lake Morphometry in Precambrian Shield Lakes. Canadian Journal of Fisheries and Aquatic Sciences.

[ref-28] Kuczyńska-Kippen N (2020). Biodiversity of zooplankton in polish small water bodies. Handbook of Environmental Chemistry.

[ref-29] Legendre P, Gallagher ED (2001). Ecologically meaningful transformations for ordination of species data. Oecologia.

[ref-30] Lévesque D, Pinel-Alloul B, Méthot G, Steedman R (2017). Effects of climate, limnological features and watershed clearcut logging on long-term variation in zooplankton communities of Boreal Shield lakes. Water.

[ref-31] Li Y, Li D, Ren B, Hu J, Li B, Krzton A, Li M (2014). Differences in the activity budgets of yunnan snub-nosed monkeys (rhinopithecus bieti) by age-sex class at xiangguqing in baimaxueshan nature reserve, China. Folia Primatologica.

[ref-54] Liu P, Xu S, Lin J, Li H, Lin Q, Han BP (2020). Urbanization increases biotic homogenization of zooplankton communities in tropical reservoirs. Ecological Indicators.

[ref-33] Liu S, Lu T, Yang D, Ren G, He X, Yang W, Cai Q, Xiao W (2018). Spatiotemporal environmental heterogeneity of alpine micro-waterbodies. Fresenius Environmental Bulletin.

[ref-34] Mackinnon J, Sha M, Cheung C, Carey G, Xiang Z, Melville D (1996). A biodiversity review of China.

[ref-35] MacLeod J, Keller W, Paterson AM (2018). Crustacean zooplankton in lakes of the far north of Ontario. Canada Polar Biology.

[ref-36] Melo TX, Medeiros ES (2013). Spatial distribution of zooplankton diversity across temporary pools in a semiarid intermittent river. International Journal of Biodiversity.

[ref-37] Oertli B, Biggs J, Céréghino R, Grillas P, Joly P, Lachavanne JB (2005). Conservation and monitoring of pond biodiversity1: introduction. Aquatic Conservation: Marine and Freshwater Ecosystems.

[ref-38] Oksanen J, Blanchet FG, Friendly M, Kindt R, Legendre P, McGlinn D, Minchin PR, O’Hara RB, Simpson GL, Solymos P, Stevens MH (2019). Vegan: community ecology package. R package version 2.5-5. https://cran.r-project.org/web/packages/vegan/index.html.

[ref-39] Pan J, Xiong F, Li W, Li A (2009). Community structure and spatial distribution of crustacean zooplankton in Lake Fuxian, Yunnan, China. Journal of Lake Sciences.

[ref-40] Pinel-Alloul B, Mimouni EA (2013). Are cladoceran diversity and community structure linked to spatial heterogeneity in urban landscapes and pond environments?. Hydrobiologia.

[ref-41] Pourriot R, Rougier C, Miquelis A (1997). Origin and development of river zooplankton: example of the Marne. Hydrobiologia.

[ref-42] R Development Core Team (2020). R: a language and environment for statistical computing. R Foundation for Statistical Computing, Vienna, Austria. https://www.R-project.org/.

[ref-43] Sagrario G, De Los Angeles M, Balseiro E, Ituarte R, Spivak E (2009). Macrophytes as refuge or risky area for zooplankton: a balance set by littoral predacious macroinvertebrates. Freshwater Biology.

[ref-44] Scheffer M, Zimmer K, Jeppesen E, Søndergaard M, Butler MG, Hanson MA, Declerck S, De Meester L (2006). Small habitat size and isolation can promote species richness: second-order effects on biodiversity in shallow lakes and ponds. Oikos.

[ref-45] Shen JR (1979). Fauna sinica crustacea freshwater copepoda.

[ref-46] Shurin JB, Winder M, Adrian R, Keller WB, Matthews B, Paterson AM, Paterson MJ, Pinel-Alloul B, Rusak JA, Yan ND (2010). Environmental stability and lake zooplankton diversity-contrasting effects of chemical and thermal variability. Ecology Letters.

[ref-47] Sommer U, Gliwicz ZM, Lampert W, Duncan A (1986). The PEG-model of seasonal succession of planktonic events in fresh waters. Archiv für Hydrobiologie.

[ref-48] Trizzino M, Bisi F, Maiorano L, Martinoli A, Petitta M, Preatoni DG, Audisio P (2014). Mapping biodiversity hotspots and conservation priorities for the Euro-Mediterranean headwater ecosystems, as inferred from diversity and distribution of a water beetle lineage. Biodiversity and Conservation.

[ref-49] Wei W, Chen R, Wang L, Fu L (2017). Spatial distribution of crustacean zooplankton in a large river-connected lake related to trophic status and fish. Journal of Limnology.

[ref-50] Williams P, Whitfield M, Biggs J, Bray S, Fox G, Nicolet P, Sear D (2004). Comparative biodiversity of rivers, streams, ditches and ponds in an agricultural landscape in Southern England. Biological Conservation.

[ref-51] Xu J, Wilkes A (2004). Biodiversity impact analysis in northwest Yunnan, Southwest China. Biodiversity and Conservation.

[ref-52] Yang W, Deng D, Zhang S, Hu C (2014). Seasonal dynamics of crustacean zooplankton community structure in Erhai Lake, a plateau lake, with reference to phytoplankton and environmental factors. Chinese Journal of Oceanology and Limnology.

