# Supplementary material for: Species diversity and community structure of crustacean zooplankton in the highland small waterbodies in Northwest Yunnan, China
Source: PeerJ. 2021 Sep 2;9:e12103. doi: 10.7717/peerj.12103 (PMC8418800; doi:10.7717/peerj.12103)
Supplement: Supplemental Information 3 [file peerj-09-12103-s003.docx]

|  | Dry season | | | Rainy season | | |
| --- | --- | --- | --- | --- | --- | --- |
| Area | Mean | Maximum | minimum | Mean | Maximum | minimum |
| Acreage (m2) | 24.3 | 160.0 | 4.0 | 23.8 | 160.0 | 3.0 |
| Depth (cm) | 27.1 | 46 | 6 | 26.8 | 50.0 | 10.0 |
| WT (℃) | 12.3 | 8.9 | 5.6 | 15.6 | 15.8 | 13.9 |
| Cond (us/cm) | 7.2 | 32.7 | 3.0 | 3.6 | 7.4 | 2.5 |
| DO (mg/L) | 6.28 | 9.11 | 3.40 | 6.26 | 8.23 | 4.60 |
| pH | 8.0 | 8.9 | 5.6 | 5.60 | 8.9 | 5.2 |
| TN (mg/L) | 0.188 | 0.399 | 0.110 | 0.214 | 0.355 | 0.104 |
| NO3N (mg/L) | 0.044 | 0.131 | 0.023 | 0.081 | 0.206 | 0.050 |
| NH3N (mg/L) | 0.053 | 0.110 | 0.030 | 0.036 | 0.121 | 0.001 |
| TP (mg/L) | 0.014 | 0.036 | 0.007 | 0.013 | 0.051 | 0.002 |
| PO4P (mg/L) | 0.006 | 0.019 | 0.004 | 0.006 | 0.034 | 0.001 |
| DSi (mg/L) | 0.484 | 0.999 | 0.123 | 0.301 | 0.034 | 0.001 |
| DOC (mg/L) | 3.631 | 5.500 | 2.420 | 9.936 | 41.460 | 2.060 |
| Chla (ug/L) | 1.162 | 7.930 | 0.161 | 1.832 | 6.144 | 0.152 |

Table 1. Mean values, maximum and minimum for limnological and morphometric variables in different season.
