# Supplementary material for: Species diversity and community structure of crustacean zooplankton in the highland small waterbodies in Northwest Yunnan, China
Source: PeerJ. 2021 Sep 2;9:e12103. doi: 10.7717/peerj.12103 (PMC8418800; doi:10.7717/peerj.12103)
Supplement: Supplemental Information 4 [file peerj-09-12103-s004.docx]

| Seasons | Variables | | |
| --- | --- | --- | --- |
|  | Limnological | Spatial | Morphometric |
| Dry season | NO_3_N  WT | MEM1  MEM3  MEM5 | Area  Depth |
| Rainy season | NO_3_N  DSi  Cond  DO | MEM1  MEM2 | Area  Depth |
